# A Prognostic Nomogram Based on Log Odds of Positive Lymph Nodes to Predict Overall Survival for Non-Metastatic Bladder Cancer Patients after Radical Cystectomy

**DOI:** 10.3390/curroncol29100539

**Published:** 2022-09-23

**Authors:** Jingtian Yang, Huasheng Huang, Wenshuang Li, Shengming Ran, Jintao Hu, Yishan Zhang, Wenjie Li, Changhao Chen, Wang He

**Affiliations:** 1 Department of Urology, Sun Yat-sen Memorial Hospital, Sun Yat-sen University, Guangzhou 510289, China; yangjt29@mail2.sysu.edu.cn (J.Y.); liwenshuang2020@163.com (W.L.); ranshm@mail2.sysu.edu.cn (S.R.); hujt7@mail2.sysu.edu.cn (J.H.); zhangysh53@mail2.sysu.edu.cn (Y.Z.); liwj278@mail2.sysu.edu.cn (W.L.); 2 Guangdong Provincial Key Laboratory of Malignant Tumor Epigenetics and Gene Regulation, Sun Yat-sen Memorial Hospital, Sun Yat-sen University, Guangzhou 510289, China; 3 Guangdong Clinical Research Center for Urological Diseases, Guangzhou 510289, China; 4 Department of Urology, Houjie Hospital of Dongguan, Dongguan 523945, China; watson15@126.com

**Keywords:** bladder cancer, radical cystectomy, nomogram, overall survival

## Abstract

(1) Purpose: The purpose of this study was to evaluate the prognostic capacity of the pathological N status (pN), lymph node ratio (LNR), and the log odds of positive lymph nodes (LODDS), and to build a prognostic nomogram to predict overall survival (OS) for bladder cancer patients treated by radical cystectomy. (2) Methods: The clinical and pathological characteristics of 10,938 patients with bladder cancer were identified from the Surveillance, Epidemiology, and End Results (SEER) database from 2004 to 2017. The predictive capacity was assessed by univariate and multivariate Cox regression analyses, the area under the receiver operating characteristic curve (AUC), and C-index. Calibration curves, decision curve analysis (DCA), and risk-grouping were utilized to evaluate the predictive accuracy and discriminative ability of the nomogram. (3) Results: LODDS was an independent risk factor for bladder cancer (all *p* < 0.001) and demonstrated the highest values of C-index and AUC. The values of AUCs in the training cohort were 0.747, 0.743, and 0.735 for predicting 1-, 3-, and 5-year OS, respectively. Calibration curves and DCA curves suggested the excellent clinical application value of our nomogram. (4) Conclusions: LODDS is a better predictive indicator for bladder cancer patients compared to pN and LNR. The LODDS-incorporated nomogram has excellent accuracy and promising clinical application value for non-metastatic bladder cancer after radical cystectomy.

## 1. Introduction

Bladder cancer (BCa) is the second most common urinary cancer worldwide [1]. For organ-confined BCa, radical cystectomy (RC) is still the most effective therapeutic procedure and recommended treatment [2,3]. Despite the rapid progress in surgical techniques in recent decades, the long-term prognosis of BCa patients after RC remains unsatisfactory among all BCa patients. A large single-center study of 1054 patients undergoing RC reported that the 5- and 10-year overall survival (OS) rates were 66% and 43% [4]. The 5-year recurrence-free and cancer-specific survival rates of 888 BCa patients who underwent RC were 58% and 66%, respectively, from a multicenter database [5]. Therefore, prognosis classification will facilitate the identification of high-risk postoperative patients who should receive additional adjuvant therapy.

The tumor, node, metastasis (TNM) classification is recommended to assess the prognosis of BCa and classify the extent of cancer dissemination [2]. For BCa patients, lymphatic metastasis is associated with a poor prognosis [6,7]. Nevertheless, the pathological N status (pN) depends only on the area of the lymph nodes (LNs) and whether there are multiple lymph node metastases in the TNM system. With failure to remove sufficient lymph nodes, inaccurate staging and prognostic prediction can occur. The exact number of examined LNs (ELN) and positive LNs (PLN) are ignored, but may be essential clinicopathological information for prognosis prediction. Numerous studies conducted efforts to set up predictive models based on novel lymph node indicators. The lymph node ratio (LNR) and the log odds of positive lymph nodes (LODDS) have been proven to be efficient parameters to predict the prognosis of various cancers [8,9,10,11]. The former refers to the ratio of PLN to ELN, while the latter is the logarithm of the ratio of PLN to negative LNs. However, no predictive nomogram based on these two indicators has been set up to predict the OS of Bca patients after surgical resection. Accurate prediction of prognosis after surgery for Bca patients is essential for treatment strategy selection, disease staging, and health management.

This study tried to assess the predictive value of pN, LNR, and LODDS, and set up a nomogram for predicting the OS of nonmetastatic BCa patients after surgical resection.

## 2. Materials and Methods

### 2.1. Patient Selection

The Surveillance, Epidemiology, and End Results (SEER) database is a publicly available cancer data collaboration program collecting clinical and pathological information on patients with cancer. The data are available via SEER*Stat software (version 8.4.0). The clinical and pathological data of patients with BCa were extracted. The inclusion criteria included (1) patients diagnosed with BCa from 2004 to 2017; (2) patients who received RC. The exclusion criteria were as follows: (1) the exact numbers of ELN and PLN were unknown; (2) the clinical and pathological data (TNM stage/treatment/survival/grade/tumor size) were incomplete or unknown; (3) patients with metastatic BCa were excluded; (4) non-urothelial carcinoma was excluded.

### 2.2. Characteristics and Identification of Independent Prognostic Factors

The clinical and pathological characteristics included age, sex, race, primary site, pathological grade, neoadjuvant chemotherapy, tumor size, T stage, pN, ELN, PLN, LNR, and LODDS. For grade, ‘well differentiated’ and ‘moderately differentiated’ were defined as ‘low grade’, while ‘poorly differentiated’ and ‘undifferentiated; anaplastic’ were defined as ‘high grade’. LNR is the ratio of PLN to ELN. LODDS was computed by the following formula: log (PLN+0.5)/(ELN-PLN+0.5). In addition, 0.5 was used to avoid an unlimited value [10,12]. LNR was divided into three subgroups, including LNR1 (<0.02), LNR2 (0.02–0.25), and LNR3 (>0.25). According to the cutoffs of −1.6 and −0.8, LODDS was divided into LODDS1 (<−1.6), LODDS2 (−1.6 ≥ LODDS ≤ −0.8), and LODDS3 (>−0.8).

The main endpoint of the current study was OS. A univariate and multivariate Cox regression model was performed to screen the independent prognostic factors. The predictive efficiency of pN, LNR, and LODDS was assessed using Kaplan–Meier curves, univariate and multivariate Cox models, the Harrell concordance index (C-index), and the area under the receiver operating characteristic curve (AUC).

### 2.3. Establishment, Validation, and Assessment of Prognostic Nomogram

The overall cohort was randomly divided into training and validation cohorts at the ratio of 7:3. Demographic, clinical, and pathological variables were compared between the two cohorts.

The significant variables from the univariate and multivariate Cox regression model were enrolled to establish the nomogram for predicting the OS of BCa patients after RC. The predictive capability of the nomogram was assessed by AUC and calibration curves. We divided participants in the overall cohort into high-risk and low-risk groups based on their total points calculated by our nomogram. The function ‘surv_cutpoint’ from R package ‘survminer’ was carried out to determine the optimal cut-point of total points. We used the decision curve analysis (DCA) and Kaplan–Meier curve based on risk subgroups to evaluate the clinical applicability of the nomogram.

### 2.4. External Validation of the Nomogram

The external validation was performed using a cohort of patients from Sun Yat-sen Memorial Hospital. We reviewed all patients diagnosed with bladder cancer from January 2013 to April 2019 retrospectively. Finally, we included 239 patients who met the inclusion criteria and exclusion criteria for external validation. AUC and risk-grouping were used to verify the accuracy and discriminative ability of the nomogram.

### 2.5. Statistical Analysis

**Figure 1:**
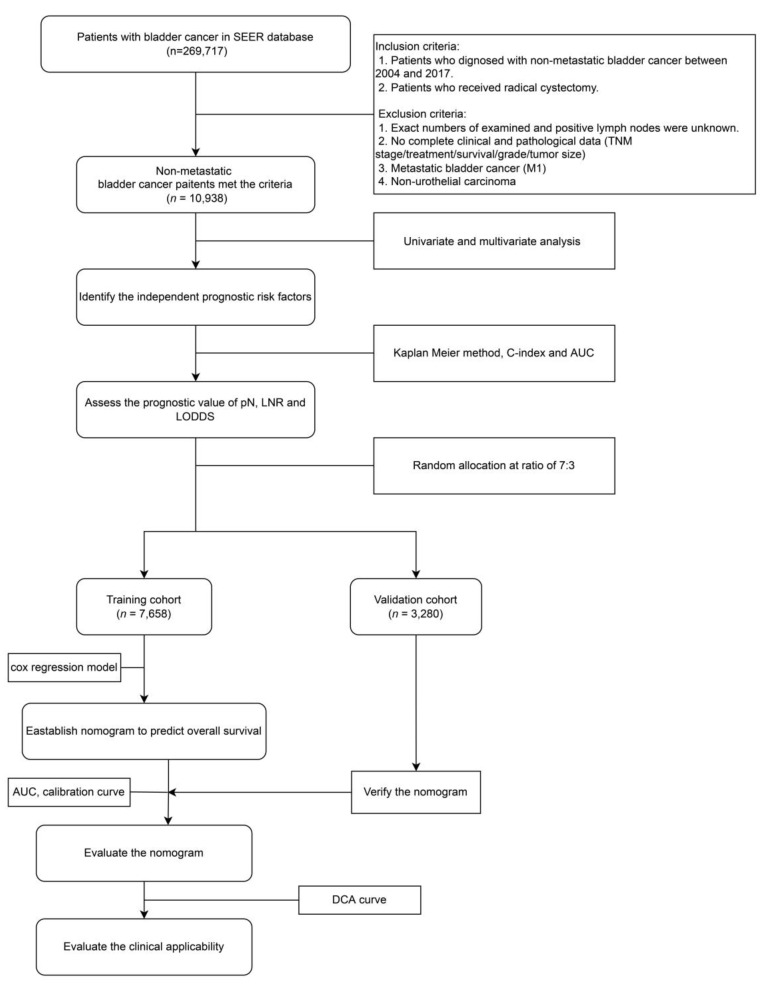
Flow chart of the study. SEER, Surveillance, Epidemiology and End Results; pN: pathological N status; LNR: lymph node ratio; LODDS: log odds of positive lymph nodes; AUC: the area under the receiver operating characteristic curve; DCA: decision curve analysis.

The data process flowchart is presented in Figure 1. All the categorical variables and the continuous variables were compared by chi-square test and Student’s t-test, respectively. Survival analysis was carried out via Kaplan–Meier curves with the Log-rank test. A *p*-value < 0.05 was considered to denote statistical significance. All the statistical tests were two-sided. All the statistical analyses were conducted with the statistical software R 4.1.3 (http://www.r-project.org; accessed on 17 April 2022).

## 3. Results

### 3.1. Clinical Characteristics

A total of 10938 bladder cancer patients were enrolled from the SEER database and randomly assigned to training (*n* = 7658) and validation cohorts (*n* = 3280) at a ratio of 7:3 randomly. Demographic, clinical, and pathological characteristics of the two cohorts were compared and are demonstrated in Table 1. In this study, 2354 patients received additional adjuvant chemotherapy. No significant difference was found in terms of demographic, clinical, and pathological characteristics between the two cohorts.

### 3.2. Survival Outcome and Independent Risk Factors

**Figure 2:**
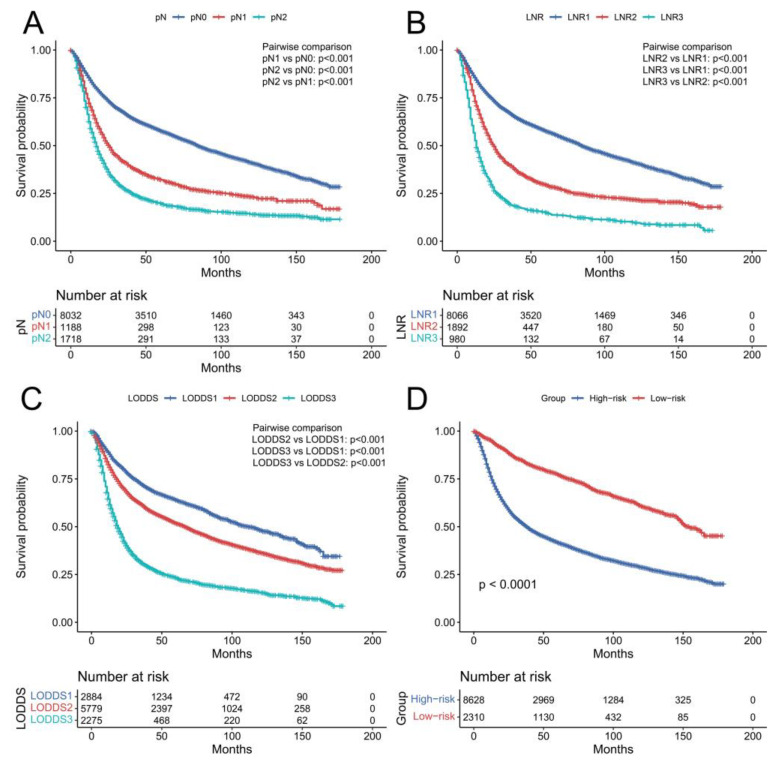
Kaplan–Meier curves of overall survival in the overall cohort according to pN (**A**), LNR (**B**), LODDS (**C**), and risk group (**D**). pN: pathological N status; LNR: lymph node ratio; LODDS: log odds of positive lymph nodes.

As shown in Figure 2, statistically significant differences in the Kaplan–Meier curves were observed among different pN, LNR, and LODDS subgroups (all *p* < 0.001). The outcomes of univariate and multivariate Cox regression analysis of OS are listed in Table 2. Univariate analysis showed that age, race, primary site, neoadjuvant chemotherapy, tumor size, T stage, pN, LNR, and LODDS were related to OS (*p* < 0.05). All these significant variables were enrolled in the multivariate analysis. According to the results of multivariate analysis, finally, age, neoadjuvant chemotherapy, tumor size, T stage, LNR, and LODDS were the independent risk factors for OS among BCa patients after surgical resection (all *p* < 0.05). In the multivariate analysis, only LNR3 predicted decreased OS (LNR2 vs. LNR1, *p* = 0.051; LNR3 vs. LNR1, *p* = 0.003). However, LODDS2 and LODDS3 were both associated with poor OS compared with LODDS1 (both *p* < 0.001).

The predictive capacity of pN, LNR and LODDS is shown in Table 3. The C-index of values of pN, LNR, and LODDS were 0.601, 0.604, and 0.608, respectively. Moreover, the AUCs of LODDS (AUC = 0.633, 0.643, and 0.638) for 1-, 3-, and 5-year OS were higher than those for pN (AUC = 0.623, 0.641, and 0.632) and LNR (AUC = 0.628, 0.643, and 0.632).

### 3.3. Establishment, Validation, and Assessment of Prognostic Nomogram

**Figure 3:**
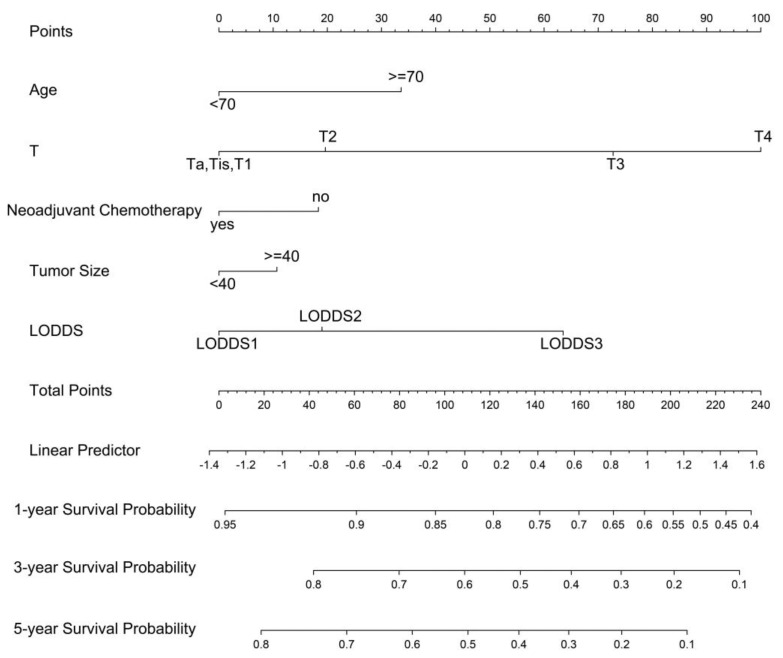
A nomogram to predict 1-, 3-, and 5- year overall survival for non-metastatic bladder cancer after surgical resection. LODDS: log odds of positive lymph nodes.

According to the results of univariate and multivariate analysis, C-index, and AUC, LODDS was incorporated into the nomogram, rather than pN and LNR. In addition to LODDS, significant independent factors in Cox regression analyses, including age, neoadjuvant chemotherapy, tumor size, and T stage were incorporated into the prognostic nomogram (Figure 3). The total score was the sum of the corresponding scores of each variable and was used to calculate the probability of OS rates.

**Figure 4:**
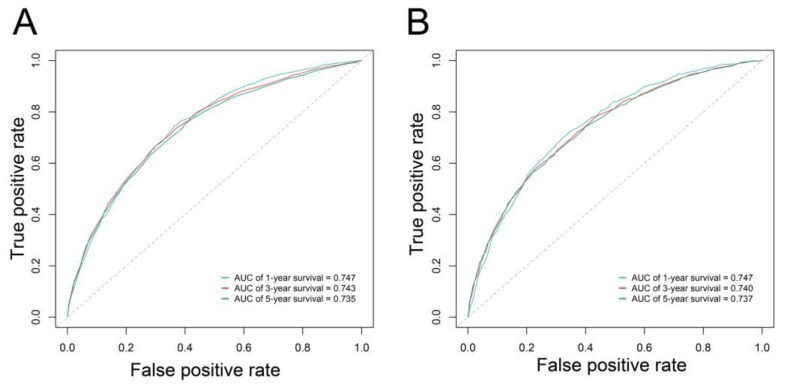
Receiver operating characteristic curve of the nomogram in the training (**A**) and validation (**B**) cohorts.

**Figure 5:**
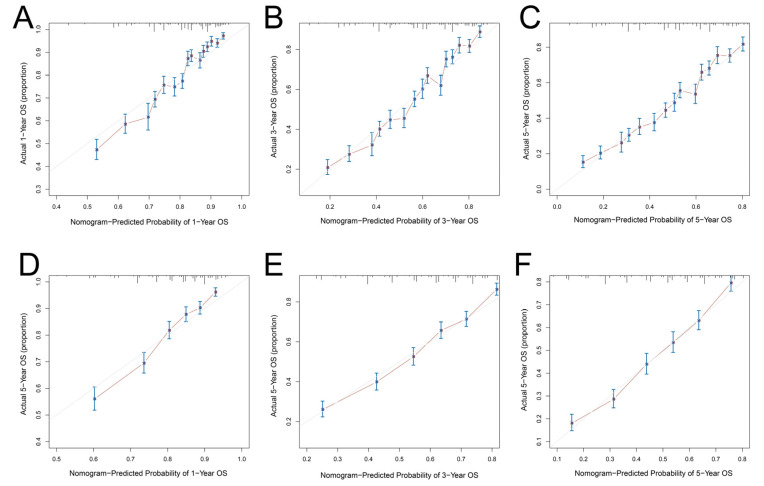
Calibration curves of the nomogram for predicting overall survival: (**A**) at 1 year in the training cohort; (**B**) at 3 years in the training cohort; (**C**) at 5 years in the training cohort; (**D**) at 1 year in the validation cohort; (**E**) at 3 years in the validation cohort; (**F**) at 5 years in the validation cohort. OS: overall survival.

**Figure 6:**
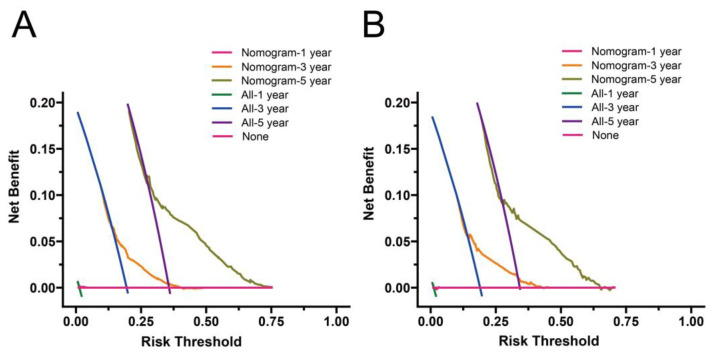
Decision curve analysis for the nomogram in the training (**A**) and validation (**B**) cohorts.

The prognostic value of the nomogram was estimated via operating characteristic curves (ROC) (Figure 4). In the training cohort, the AUCs of the 1-, 3-, and 5-year OS rates were 0.747, 0.743, and 0.735, while in the validation cohort the AUCs were 0.747, 0.740, and 0.737, respectively. The ROC curves show the excellent predictive accuracy of the nomogram. In the calibration curves, the good accordance between the predicted and actual OS rates was shown for the training cohort and validation cohort (Figure 5). The DCA curves demonstrated the optimal clinical application value (Figure 6). The optimal cut-point of total points for risk-grouping was 76.28. After subgroup analysis, we found that high-risk bladder cancer patients had a decreased OS (*p* < 0.001), which demonstrated the excellent discriminating power of our nomogram (Figure 2D).

**Figure 7:**
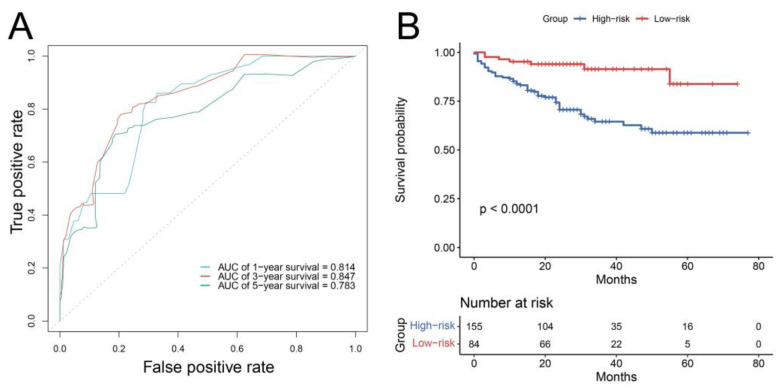
Receiver operating characteristic curve (**A**) and Kaplan–Meier curves of risk group (**B**) of the external validation cohort.

Furthermore, the predictive performance of the nomogram was validated externally by the ROC curve and Kaplan–Meier curves of risk groups. In the external cohort, the AUCs for the 1-, 3-, and 5-year OS rates were 0.814, 0.847, and 0.783 (Figure 7A). After risk grouping, we found that high-risk bladder cancer patients had a decreased OS compared to low-risk patients (*p* < 0.001), indicating good discriminating power of our nomogram (Figure 7B).

## 4. Discussion

Several studies have noted the importance and clinical value of novel lymph node indicators including LNR and LODDS for prognostic prediction in various cancers [8,9,10,11]. In our research, we evaluated the predictive ability of pN, LNR, and LODDS, and then established a nomogram based on the most effective lymph node indicator of OS for BCa patients after surgical resection within a large cohort. Excellent discrimination and calibration were presented and validated in the prognostic model by the ROC curves and calibration curves. Subgroup Kaplan–Meier analysis according to risk group also showed that the nomogram can identify high-risk bladder cancer patients with poor prognostic outcomes after surgical resection.

Several studies have attempted to evaluate the prognostic ability of novel indicators, including LNR and LODDS. Analysis of data from two prestigious cancer centers showed that pN and LNR were significant predictors for disease-specific survival (*p* < 0.1), but only LNR higher than 20% was associated with decreased disease-specific survival [13]. Comparable results were presented by Fleischmann et al., who analyzed a consecutive cohort of 507 patients with urothelial carcinoma of bladder cancer [14]. In the univariate analysis, different LNRs had a significant influence on recurrence-free survival (*p* = 0.0034) and OS (*p* = 0.0002) [14]. A mate-analysis with a total number of 3311 patients across 14 studies also reported similar results [15]. In addition to LNR, LODDS was considered to be a more accurate indicator for the lymph node category in various studies. A retrospective study had shown that LODDS was a superior predictive indicator for disease-free survival compared to pN and LNR in patients with rectal cancer [16]. Similar results and conclusions can be found in various studies with different cancers including lung squamous cell carcinoma [17], squamous cell carcinoma of the penis [18], colon cancer [19], endometrial carcinosarcoma [20], etc. For bladder cancer, Jin et al. assessed the predictive capacity of pN, LNR, and LODDS in patients with muscle-invasive urothelial carcinoma of bladder cancer [12]. They found that LODDS was better at predicting prognosis for muscle-invasive urothelial carcinoma of bladder cancer compared with pN and LNR [12]. However, the population of this study was limited to muscle-invasive bladder cancer patients, and histology was confined to transitional cell carcinoma. We compared the predictive ability of three lymph node indicators in non-metastatic bladder cancer patients and all pathological types were incorporated. In addition, a predictive nomogram for wider stage and pathological types was set up to predict OS for bladder cancer patients.

The nomogram incorporated several independent prognostic factors from univariate and multivariate analyses, including age, neoadjuvant chemotherapy, tumor size, and T stage. In previous studies, age and T stage have always been the independent risk factors for survival outcomes [21,22,23]. Meanwhile, tumor size has been considered as an independent risk factor of OS for patients after radical cystectomy in previous studies [24,25,26]. Moreover, mounting evidence has proven that neoadjuvant chemotherapy followed by radical cystectomy improves the survival outcome compared to radical cystectomy alone [27,28,29].

To our knowledge, for the first time, this study developed a nomogram incorporating LODDS and confirmed that LODDS is a valuable predictive factor for the overall survival of bladder cancer for the first time. Furthermore, the establishment of our prognostic model was based on a very large cohort from the SEER database, which provided large amounts of clinicopathological data with credibility. The accuracy and clinical application value of our prognostic model were demonstrated, and the clinicopathological variables incorporated in our nomogram can be readily obtained in clinical practice. Moreover, a visible nomogram can make it easier for clinicians to estimate 1-, 3-, and 5-year OS for BCa patients, which can assist in the development of individualized treatment strategies and health management. Moreover, the risk subgrouping based on the prognostic model allows clinicians to identify high-risk patients with worse prognoses and develop a more aggressive treatment plan.

We should point out several limitations of our study. It was a retrospective study, although the current research was based on a large cohort of the population. Further validation of our findings is recommended prospectively in a multi-center context. Moreover, the data regarding cancer specific survival were not analyzed or described in this study. In addition, some clinical information could not be extracted from the SEER database, such as the chemotherapy regimen and the position of positive lymph nodes. More valuable clinicopathological information should be collected to refine our prognostic model.

## 5. Conclusions

For postoperative patients with bladder cancer, LODDS is a valuable prognostic factor with an excellent predictive ability compared to pN and LNR. A prognostic nomogram based on a large cohort of the population was established, incorporating age, neoadjuvant chemotherapy, tumor size, T stage, and LODDS. This prognostic model may facilitate the prediction of prognosis and individual treatment strategy development for clinicians.

## Figures and Tables

**Table 1:** Clinical and pathological characteristic.

Variables	Overall Cohort	Training Cohort	Validation Cohort	*p*
	(*n* = 10,938)	(*n* = 7658)	(*n* = 3280)	
Age, *n* (%)				1.000
<70	5889 (53.8%)	4123 (37.7%)	1766 (16.1%)	
≥70	5049 (46.2%)	3535 (32.3%)	1514 (13.8%)	
Sex, *n* (%)				0.139
Female	2658 (24.3%)	1830 (16.7%)	828 (7.6%)	
Male	8280 (75.7%)	5828 (53.3%)	2452 (22.4%)	
Race, *n* (%)				0.856
White	9623 (88%)	6734 (61.6%)	2889 (26.4%)	
Others/Unknown	1315 (12%)	924 (8.4%)	391 (3.6%)	
Primary site, *n* (%)				0.362
Trigone of bladder	690 (6.3%)	495 (4.5%)	195 (1.8%)	
Lateral wall of bladder	1924 (17.6%)	1365 (12.5%)	559 (5.1%)	
Posterior wall of bladder	874 (8%)	621 (5.7%)	253 (2.3%)	
Others/Unknown	7450 (68.1%)	5177 (47.3%)	2273 (20.8%)	
Grade, *n* (%)				0.428
Low grade	337 (3.1%)	243 (2.2%)	94 (0.9%)	
High grade	10601 (96.9%)	7415 (67.8%)	3186 (29.1%)	0.561
Neoadjuvant chemotherapy, *n* (%)				
Yes	1937 (17.7%)	1345 (12.3%)	592 (5.4%)	
No	9001 (82.3%)	6313 (57.7%)	2688 (24.6%)	
Tumor size, *n* (%)				0.967
<40 mm	5364 (49%)	3754 (34.3%)	1610 (14.7%)	
≥40 mm	5574 (51%)	3904 (35.7%)	1670 (15.3%)	
T, *n* (%)				0.882
Ta,Tis,T1	1176 (10.8%)	824 (7.5%)	352 (3.2%)	
T2	4051 (37%)	2836 (25.9%)	1215 (11.1%)	
T3	3991 (36.5%)	2781 (25.4%)	1210 (11.1%)	
T4	1720 (15.7%)	1217 (11.1%)	503 (4.6%)	
pN, *n* (%)				0.622
pN0	8032 (73.4%)	5619 (51.4%)	2413 (22.1%)	
pN1	1188 (10.9%)	822 (7.5%)	366 (3.3%)	
pN2	1718 (15.7%)	1217 (11.1%)	501 (4.6%)	
Examined lymph nodes, mean ± SD	18.32 ± 14.66	18.43 ± 14.72	18.04 ± 14.53	0.205
Positive lymph nodes, mean ± SD	0.88 ± 2.5	0.91 ± 2.59	0.81 ± 2.3	0.069
LNR, *n* (%)				0.916
LNR1	8066 (73.7%)	5648 (51.6%)	2418 (22.1%)	
LNR2	1892 (17.3%)	1329 (12.2%)	563 (5.1%)	
LNR3	980 (9%)	681 (6.2%)	299 (2.7%)	
LODDS, *n* (%)				0.629
LODDS1	2884 (26.4%)	2005 (18.3%)	879 (8%)	
LODDS2	5779 (52.8%)	4069 (37.2%)	1710 (15.6%)	
LODDS3	2275 (20.8%)	1584 (14.5%)	691 (6.3%)	

pN: pathological N status; LNR: ratio of lymph node ratio; LODDS: log odds of positive lymph nodes; SD: standard deviation.

**Table 2:** Prognostic factors for overall survival of patients.

Variable	Univariate Analysis			Multivariate Analysis		
	HR	95% CI	*p* Value	HR	95% CI	*p* Value
Age, *n* (%)						
<70	Reference			Reference		
≥70	1.620	1.54–1.7	<0.001	1.580	1.5–1.67	<0.001
Sex, *n* (%)						
Male	Reference					
Female	1.050	0.99–1.11	0.111			
Race, *n* (%)						
White	Reference			Reference		
Others/Unknown	1.090	1.01–1.17	0.036	1.070	0.99–1.15	0.101
Primary site, *n* (%)						
Trigone of bladder	Reference			Reference		
Lateral wall of bladder	0.810	0.72–0.91	0.001	0.940	0.84–1.06	0.339
Posterior wall of bladder	0.890	0.78–1.02	0.103	0.940	0.82–1.07	0.353
Others/Unknown	1.010	0.92–1.12	0.782	1.040	0.93–1.15	0.497
Grade, *n* (%)						
Low grade	Reference			-	-	-
High grade	1.080	0.94–1.25	0.283	-	-	-
Neoadjuvant chemotherapy, *n* (%)						
Yes	Reference			Reference		
No	1.410	1.3–1.52	<0.001	1.220	1.13–1.32	<0.001
Tumor size, *n* (%)						
<40 mm	Reference			Reference		
≥40 mm	1.300	1.24–1.37	<0.001	1.170	1.11–1.23	<0.001
T, *n* (%)						
Ta,Tis,T1	Reference			Reference		
T2	1.340	1.2–1.5	<0.001	1.300	1.16–1.46	<0.001
T3	2.910	2.61–3.25	<0.001	2.320	2.08–2.6	<0.001
T4	4.120	3.67–4.62	<0.001	3.030	2.69–3.42	<0.001
pN, *n* (%)						
pN0	Reference			Reference		
pN1	1.910	1.77–2.06	<0.001	0.800	0.48–1.34	0.401
pN2	2.720	2.55–2.89	<0.001	0.830	0.5–1.4	0.492
LNR, *n* (%)						
LNR1	Reference			Reference		
LNR2	1.980	1.86–2.1	<0.001	1.670	1–2.79	0.051
LNR3	3.460	3.21–3.73	<0.001	2.210	1.31–3.73	0.003
LODDS, *n* (%)						
LODDS1	Reference			Reference		
LODDS2	1.450	1.35–1.55	<0.001	1.220	1.13–1.31	<0.001
LODDS3	3.080	2.86–3.32	<0.001	1.580	1.43–1.74	<0.001

HR: hazard ratio; CI: confidence interval; pN: pathological N status; LNR: lymph node ratio; LODDS: log odds of positive lymph nodes.

**Table 3:** Evaluation of the prognostic value of pN, LNR, and LODDS.

	C-Index	AUC		
		1-Year OS	3-Year OS	5-Year OS
pN	0.601	0.623	0.641	0.632
LNR	0.604	0.628	0.643	0.632
LODDS	0.608	0.633	0.643	0.638

AUC: the area under the receiver operating characteristic curve; pN: pathological N status; LNR: lymph node ratio; LODDS: log odds of positive lymph nodes; OS: overall survival.

## Data Availability

Publicly available datasets were analyzed in this study. These data can be found here: https://seer.Cancer.gov/ (accessed on 19 February 2022).
